# The Role of MicroRNAs in Muscle Tissue Development in Beef Cattle

**DOI:** 10.3390/genes11030295

**Published:** 2020-03-11

**Authors:** Sayed Haidar Abbas Raza, Nurgulsim Kaster, Rajwali Khan, Sameh A. Abdelnour, Mohamed E. Abd El-Hack, Asmaa F. Khafaga, Ayman Taha, Husein Ohran, Ayman A. Swelum, Nicola M. Schreurs, Linsen Zan

**Affiliations:** 1 College of Animal Science and Technology, Northwest A&F University, Yangling 712100, Shaanxi, China; haiderraza110@nwafu.edu.cn (S.H.A.R.); kacter-83@mail.ru (N.K.); rajwalikhan@nwafu.edu.cn (R.K.); 2 Department of Animal Production, Faculty of Agriculture, Zagazig University, Zagazig 44511, Egypt; samehtimor86@gmail.com; 3 Department of Poultry, Faculty of Agriculture, Zagazig University, Zagazig 44511, Egypt; dr.mohamed.e.abdalhaq@gmail.com; 4 Department of Pathology, Faculty of Veterinary Medicine, Alexandria University, Edfina 22758, Egypt; Asmaa.Khafaga@alexu.edu.eg; 5 Department of Animal Husbandry and Animal Wealth Development, Faculty of Veterinary Medicine, Alexandria University, Edfina 22578, Egypt; Ayman.Taha@alexu.edu.eg; 6 Department of Physiology, University of Sarajevo, Veterinary Faculty, Zmaja od Bosne 90, 71 000 Sarajevo, Bosnia and Herzegovina; husein.ohran@vfs.unsa.ba; 7 Department of Theriogenology, Faculty of Veterinary Medicine, Zagazig University, Zagazig 44511, Egypt; aymanswelum@zu.edu.eg; 8 Animal Science, School of Agriculture and Environment, Massey University, Palmerston North 4442, New Zealand; N.M.Schreurs@massey.ac.nz; 9 National Beef Cattle Improvement Center, Northwest A&F University, Yangling 712100, Shaanxi, China

**Keywords:** microRNA, meat quality, carcass, development, cattle

## Abstract

In this review, we highlight information on microRNA (miRNA) identification and functional characterization in the beef for muscle and carcass composition traits, with an emphasis on Qinchuan beef cattle, and discuss the current challenges and future directions for the use of miRNA as a biomarker in cattle for breeding programs to improve meat quality and carcass traits. MicroRNAs are endogenous and non-coding RNA that have the function of making post-transcriptional modifications during the process of preadipocyte differentiation in mammals. Many studies claim that diverse miRNAs have an impact on adipogenesis. Furthermore, their target genes are associated with every phase of adipocyte differentiation. It has been confirmed that, during adipogenesis, several miRNAs are differentially expressed, including miR-204, miR-224, and miR-33. The development of mammalian skeletal muscle is sequentially controlled by somite commitment into progenitor cells, followed by their fusion and migration, the proliferation of myoblasts, and final modification into fast- and slow-twitch muscle fibers. It has been reported that miRNA in the bovine MEG3-DIO3 locus has a regulatory function for myoblast differentiation. Likewise, miR-224 has been associated with controlling the differentiation of bovine adipocytes by targeting lipoprotein lipase. Through the posttranscriptional downregulation of KLF6, miR-148a-3p disrupts the proliferation of bovine myoblasts and stimulates apoptosis while the miR-23a~27a~24-2 cluster represses adipogenesis. Additional to influences on muscle and fat, bta-mir-182, bta-mir-183, and bta-mir-338 represent regulators of proteolysis in muscle, which influences meat tenderness.

## 1. Introduction

Globally, cattle are the most commonly farmed animal and many cattle breeds have developed for different purposes, including milk, meat, and hide production, as well as for draught purposes. In China, the Qinchuan breed is used for beef production and is particularly produced on the Guanzhong Plain in the Shaanxi province, but is also used in other provinces in China [1].

In the cell, microRNA (miRNA) is found extra- and intra-cellularly and are non-coding RNA, which are approximately 22 nucleotides long. miRNA post-transcriptionally regulates protein expression and is associated with normal and diseased cells. The miRNA work on cellular processes associated with cell development and differentiation, immunity, reproduction, and metabolism [2,3,4,5]. 

The miRNA molecules have a conserved biogenesis and target sites at the three prime untranslated regions (3’UTRs) of eukaryotic mRNAs [2]. miRNA target site mutations have been associated with many complex phenotypes in humans, domestic animals, and plants [6,7,8]. The development of the characterization and sequencing techniques of miRNAs has not only revealed their functions in several cellular processes, but also showed abnormalities in miRNA expression in various phenotypes [9]. In this paper, we review the available data on miRNA identification and function on carcass and muscle traits for bovines, with an emphasis on Qinchuan beef cattle, and discuss the current challenges and future directions for the use of miRNAs in cattle. 

## 2. Determinant Factors in Meat Tenderness as a Quality Trait

**Figure 1:**
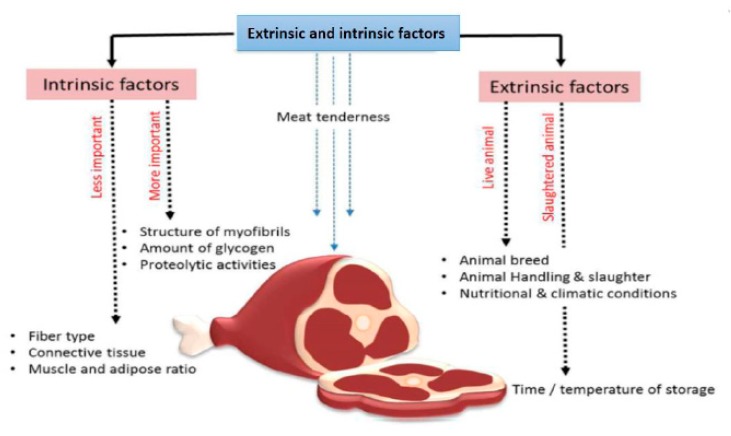
Extrinsic and intrinsic factors affecting meat tenderness.

When purchasing meat products, modern consumers make decisions based on considering quality against price. Historically, the selection of animals for meat production has mainly focused on yield with less of a focus on meat quality. Meat tenderness is a critical quality trait resulting from diverse factors; it is a product of the animal’s genetic potential and various environmental factors To improve meat tenderness, the study of both extrinsic and intrinsic factors is required as together they influence the resulting characteristics of the meat (Figure 1). Researching these two associated factors and their impact on meat quality will ultimately lead to improvements in beef.

### 2.1. Extrinsic and Intrinsic Factors

Extrinsic factors that affect meat tenderness include feeding practices, handling of animals at slaughter, and also the time and temperature for cooling and storing the carcass (Figure 1). Handling conditions of the carcass, including things as simple as the way beef hangs after slaughter, influence the tension exerted on different muscles, which directly influences the degree of meat tenderness [10]. The intrinsic characteristics of muscle meat, such as fiber type, the structure of the myofibrils, connective tissue, muscle and adipose ratio, amount of glycogen, and proteolytic activities in the muscle, are all traits that determine meat tenderness (Figure 1).

### 2.2. miRNAs and Muscle System Regulation

In cattle, miRNAs have been widely described to be key regulators of skeletal muscle traits (Table 1). For instance, in miRNA functional studies of skeletal muscle development in calves, miR-148a-3p has the function of inhibiting the proliferation of bovine muscle cells and promotes apoptosis [11]. In a comparison of Holstein–Friesian and Hereford/Limousine breeds, miRNA expression, profiled using microarray, found nine miRNAs related to muscle cell differentiation and development [12]. In Chinese Qinchuan cattle, 104 novel and 417 known miRNAs were discovered, and five muscle-specific miRNAs were identified: bta-miRNA-206, miRNA-1, miRNA-133, miRNAn12, and miRNAn17, which had high expression levels in muscle or organs linked to muscle [13]. The growth of muscle tissue, meat quality, and muscle-linked diseases can directly be associated with the differentiation of skeletal muscle-derived satellite cells (MDSC) in cattle. Zhang et al. [14] showed that the differentiation of MDSC in cattle was associated with the expression of 564 known and 53 novel miRNAs [14]. Wang et al. [15] recognized that myocyte enhancer factor 2A (MEF2A) controlled skeletal myoblast differentiation through regulating protein phosphatase 2A (PP2A) signaling and the maternally expressed 3 (MEG3)–iodothyronine deiodinase 3 (DIO3) miRNA mega cluster. The authors reported that MEF2A was sufficient to induce the expression of MEG3 in bovine skeletal myoblasts. MEF2A regulated PP2A signaling through protein phosphatase 2 regulatory subunit B, gamma (PPP2R2C) during bovine myoblast differentiation [15]. Additionally, expression of miR-543 and miR-758 supported myoblast differentiation and inhibited the expression of PPP2R2C. These marks indicate that the MEG3–DIO3 miRNAs function at MEF2A downstream to modulate the PP2A signaling during myoblast differentiation in bovines [15]. During muscle development, Peng et al. [16] considered that the overexpression of miR-744 may promote cell proliferation and suppress myoblast apoptosis [16]. It was noticeable that the sequence of miR-744 is highly conserved across species such as cow, human, and mouse.

Romao et al. [17] revealed that intronic miRNAs, miR-33a and miR-1281, had synchronized expression levels according to their host genes that are involved in lipid metabolism, including with transcriptional factor SREBF2 and EP300, which is a co-activator of transcriptional factor C/EBPα [17]. The results indicate that these factors have important regulative roles in lipid metabolism (lipogenesis and lipolysis) and adipogenesis (formation of adipose tissue from precursor cells) in cattle. Zhang et al. [18] claim that miR-224, by targeting lipoprotein lipase (LPL), is associated with preadipocyte differentiation in bovines, and provides a better understanding of the molecular basis of intramuscular fat (IMF) accumulation [18]. Bta-miR-130 overexpression resulted in decreased triacylglycerol (TAG) values in the cell, throughout adipogenesis, and reduced the formation of body fat [19]. Bta-miR-130a/b inhibition also gave rise to greater lipid droplets, as well as the accumulation of TAG. Bta-miR-130a/b overexpression downregulated the expression levels of genes related with differentiation of adipocytes, such as C/EBPα, C/EBPβ, FABP4, PPARG, LPIN1, and LPL

**Figure 2:**
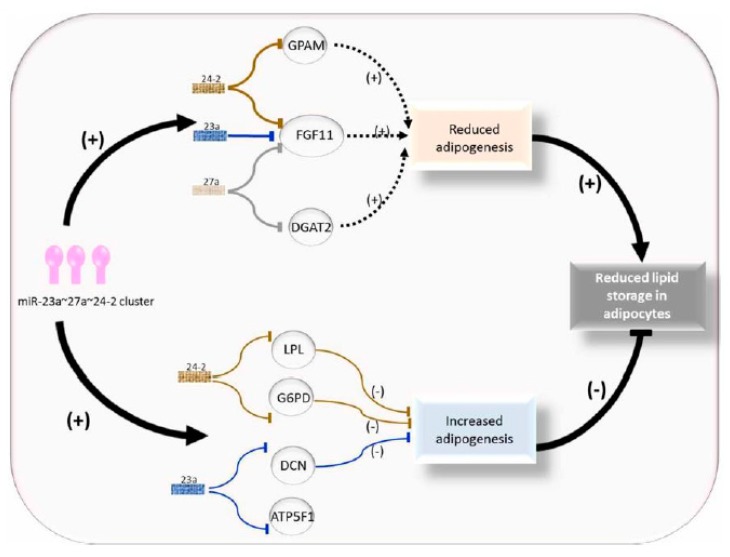
Role of the miR-23a~27a~24-2 cluster in regulating adipocyte adipogenesis.

The miR-23a~27a~24-2 cluster down regulates adipogenesis in cattle (Figure 2). In the study by Guan [20], bovine fetuses were screened using high-throughput sequencing (HTS) technology, and the data revealed bta-miR-23a acted on bovine adipogenesis through skeletal muscle as an adipogenic miRNA [20]. The expression of bta-miR-23a is associated with decreased fat aggregation and works through inhibiting the key adipogenic transcription factor peroxisome proliferative-activated receptor gamma (PPARγ) and CCAAT/enhancer binding protein alpha (C/EBPα) [20]. Kappeler et al. [21] analyzed deep sequencing miRNA libraries of longissimus thoracis (LT) muscle and identified a total of 42 novel and 308 already known miRNAs, and seven of the known miRNAs were associated with skeletal muscle tissue [21]. 

Feed efficiency (measured as residual feed intake) is one of the most important issues in beef production for minimizing the cost of production. Energy metabolism of skeletal muscle can create variations in feed efficiency [21,22]. In Nelore cattle, De Oliveira et al. [23] identified hub and differentially expressed miRNA, which were associated with variation in residual feed intake, and included bta-miR-7, bta-miR15a, bta-miR-21, bta-miR-29, bta-miR-30b, bta-miR-106b, bta-miR-199a-3p, bta-miR-204, bta-miR-296, and bta-miR-486 [23]. 

## 3. Candidate miRNAs Involved in Carcass Quality

### 3.1. miR-224 and Fat Expression

miR-224 has a role in adipogenesis, lipid metabolism [24], and adipocyte apoptosis [25] by inhibiting adipocyte differentiation and impeding apoptosis of 3T3-L1 cells [24,25]. Zhang et al. [26] found an inverse relationship between miR-224 expression and the levels of LPL, and the miR-224 expression was lower in steers, accompanied by a higher IMF content [26]. The free-binding energy (−27.75) and score (164) are greater between miR-224 and LPL compared to another microRNA. Free-binding energy indicates how a protein recognizes its biologically relevant ligand or a small molecule inhibitor and allows an understanding of the processes of biological recognition [26].

The association between the metabolism of lipids and miR-224 in cattle was clarified by targeting the LPL gene [18] and revealed that miR-224, by binding to the 1524–1527 bp part in the LPL gene (LPL 3′UTR2), suppresses the expression of LPL in 293T cells. The LPL has a vital function for adipogenic differentiation [27], which coincides with the findings of Zhang et al. [18], in that miR-224, by targeting LPL, impedes the adipogenic differentiation in bovines.

By targeting EGR2 and ACSL4 in 3T3-L1 cells, miR-224 plays a role in fatty acid metabolism and in early adipocyte differentiation [24], which proves the presence of numerous targets in the differentiation of adipocytes, regulated by miR-224. Nevertheless, a detailed description of the miR-224/c/EBPs/PPARγ axis, and its role in the regulation of these differentiation mechanisms, is not completely clear, and further research is needed.

### 3.2. The miR-23a~27a~24-2 Cluster

Generally, clustered miRNAs are expressed from the same transcript [28,29], and the host gene encoding these miRNAs possesses an intact gene structure [30]. Usually, a miRNA cluster contains several miRNAs, and the clustered miRNAs have similar but also individual functions [31]. 

It has been determined that the miR-23a~27a~24-2 cluster takes part in the regulation of endothelial cell apoptosis [32], osteoblast differentiation [33], neuronal apoptosis [34], and erythropoiesis [35]. During the differentiation of bovine adipocyte progenitor cells and 3T3-L1 cells, miR-23a serves as a negative regulator [20,36]. By targeting the peroxisome proliferator activated receptor gamma (PPAR γ), miR-27a represses 3T3-L1 preadipocyte differentiation [37], and miR-27a is also involved in the formation of brown fat [38], porcine preadipocyte differentiation [39], and high-fat diet-induced insulin resistance [40]. Recently, Wang et al. [41] found that miR-23a targets DCN, miR-24-2 targets G6PD, and LPL, and that all these three genes inhibit adipogenesis [37]. 

The miR-23a~27a~24-2 cluster has a regulatory role in bovine adipogenesis, more precisely it represses adipogenic preadipocyte differentiation in bovines. This inhibitory effect is achieved through a balanced regulation via targeting three types of genes with anti-adipogenic effects (DCN, G6PD, and LPL) and pro-adipogenic effects (GPAM, DGAT2, and FGF11 [37]. The contribution of the pro-adipogenic targets GPAM, DGAT2, and FGF11 to adipogenesis might be greater than that of DCN, G6PD, and LPL, and this may lead to the reduced adipogenesis phenotype observed in the presence of the miR-23a~27a~24-2 cluster compared to the individual miRNA [37]. Further research on the molecular basis of this cluster in adipogenesis could provide insight for selecting candidates with more intramuscular fat content. Subsequently, the miR-23a~27a~24-2 cluster can be considered as a candidate target for genomic selection breeding in beef cattle to improve the meat quality. More scientific evidence is required to clarify the exacting mechanisms underlying the cooperative roles of the miR-23a~27a~24-2 cluster related to fat deposition and meat quality in production animals.

### 3.3. MEG3–DIO3 miRNA Cluster (miR-758 and miR-543)

The MEG3–DIO3 locus in bovines has an essential regulatory function for the regeneration of skeletal muscle [42,43]. In mammals, Gtl2–Dio3 is the largest expressed miRNA cluster and is entirely expressed from the maternally inherited allele [28]. The expression pattern of these miRNAs controls the adjacent Gtl2 promoter [42,44]. In skeletal muscles, the regulatory functions exerted by this gene cluster are not totally understood. Like previously mentioned, MEF2A is important for skeletal myoblast differentiation, and its inhibition could lead to the disrupted formation of myotubes [41]. Recently, Wang et al. [15] discovered a novel regulatory molecular role of MEF2A and have proven the regulative role of MEF2A in the MEG3–DIO3 miRNA cluster, and also a role in the signaling of PP2A during the process of differentiation of myoblasts in skeletal muscle [15]. The possible function of the MEF2A–MEG3/DIO3–PP2A axis is to downstream MEF2A, in order to modulate the signaling of PP2A in the differentiation of the myoblast. The main target of the MEG3–DIO3 miRNA cluster is the PP2A subunit genes and miR-758 and miR-543 target PPP2R2C [15]. The PP2A holoenzyme includes A, B, and C subunits, which different genes encode [45]. 

Additionally, the MEF2A–MEG3/DIO3–PP2A regulatory pathway has a crucial function in the regeneration of skeletal muscle. MEF2A is an essential part of activating the regeneration of muscle [42,46].

### 3.4. Bta-miR-130

The miR-130 family includes two members with the same source sequences—miR-130a and miR-130b [47]. The miR-130 family impacts on the differentiation of adipocytes by inhibiting PPARG expression, and this inhibits adipocyte production [48]. The miR-130a/b members, which are regulated by C/EBPα, have essential functions in lipid metabolism by controlling translational levels of PPARG and PPAR α [49]. Greater expression of bta-miR-130a lowers the concentration of triglyceride in epithelial cells in mammals and also interferes with the formation of lipid droplets [50]. miR-130b inhibits the differentiation of adipocytes, by targeting PGC1 α, which is involved in regulating fat metabolism [51]. Ma et al. [19] showed that higher expression of both bta-miR-130a/b decreased the PPARG expression, while lower expression increased the PPARG expression [19]. During adipogenesis, bta-miR-130a/b inhibits the formation of lipid droplets and TAG levels. 

At the gene level, bta-miR-130a reduces the expression of genes linked to differentiation, like C/EBPα, C/EBPβ, FABP4, LPIN1, and LPL, while increasing the expression of SREBP1 [19]. Such results were compatible with Lee et al. [48], where the enhancement of adipogenesis was the result of the downregulation of miR-130, while overexpression of miR-130 disrupted the adipogenesis process. An inhibitor of Bta-miR-130a decreased the expression levels of SREBP1. The SREBP1 gene is expressed in mature white adipose tissue and has a function in adipocyte differentiation. Overexpression of miR-130a inhibited the expression levels of PPARG, suggesting that miR-130a has a role in TAG synthesis in epithelial cells through targeting PPARG in bovines [50]. PPARG, which is a part of the PPAR family, regulates the differentiation process [52]. High expression levels of bta-miR-130a/b also stimulate the CYP2U1 expression at both the mRNA and protein levels, and as CYP2U1 is capable of lowering triglyceride levels, it could be considered that CYP2U1 is also a target gene for miR-130a/b [53]. It can be concluded that bta-miR-130a/b acts as a negative regulator of adipocyte differentiation, by inhibiting the expression of PPARG, but intensifying the CYP2U1 expression.

### 3.5. miR-148a-3p

miR-148a has an influence on physiological functions like cell growth, cell death, and tumorigenesis, due to its regulatory role in cell proliferation. Concerning muscle and carcass characteristics in cattle, miR-148a, a myogenic microRNA, regulates myogenic differentiation by directly targeting the 3′-UTR of ROCK1, [54] while, miR-434-3p is an antiapoptotic miRNA, mainly located in skeletal muscles. Song et al. [11] examined expression levels of miR-148a-3p in several muscle cells in bovines, and concluded that it is well expressed in muscle tissue but showed reduced expression levels in muscle cells during the growth stages when exogenous miR-148a downregulated the expression levels of genes related to cell proliferation, like PCNA, CDK2, and cyclinD1 [11]. It was observed and can be concluded that overexpression of miR-148a-3p interferes with skeletal muscle cell proliferation in bovines [11]. Song et al. [11] identified that a possible target gene of miR-148a-3p may also be KLF6, which is very important for the development of skeletal muscles in bovines [11].

The opposing roles of miR-148a-3p in the proliferation and apoptosis of bovine muscle cells could be used to prove the hypothesis that apoptosis is used to balance the effect of cell proliferation. Due to the earlier findings that exogenous miR-148a might have a potential role in regulating the apoptosis process and cell proliferation of bovine muscle cells, there is a need for further research as to how miR-148a-3p will influence the growth, carcass characteristics, and meat quality of cattle.

### 3.6. Bta-miR-23a

Like the previously mentioned miRNAs, miR-23a has a regulatory function for adipogenesis in 3T3-L1 cells, but the exact mechanism was not completely clarified. Based on novel studies, it can be concluded that miR-23a inhibit the expression of ZNF423, acting as a negative regulator in adipogenesis in bovines. miR-23a has the capacity to target regulatory proteins that control stem cell fate during differentiation, and miR-23a has been identified as having roles in regulating both osteogenesis and chondrogenesis [55,56]. Due to the correlation between miR-23a and ZNF423, miR-23a could possibly have a regulatory function in energy metabolism and also be engrossed in the pathogenesis of some diseases related to metabolism. Another function of miR-23a is to suppress the differentiation process in the endoderm and ectoderm lineages, downregulating the differentiation of embryonic stem cells [57]. The exact function and mechanisms of miR-23a in the mentioned processes are not exactly known, and this needs further consideration for its effects on muscle growth and composition in cattle. 

### 3.7. Bta-miR-182, Bta-miR-183, and Bta-miR-338

Bta-mir-182, bta-mir-183, and bta-mir-338 regulate the expression levels of genes associated with several pathways. This review will discuss only the pathways that are related to muscle proteolysis and meat tenderness. Kappeler et al. [21] claim that MEF2C, MAP3K2, MTDH, and TNRC6B are genes that are generally targets of the three differentially expressed miRNAs [21]. Up-regulation of bta-mir-182 with EBVSF14 down-regulates the anti-apoptotic BCL2 protein, therefore promoting apoptosis that can lead to muscle proteolysis and tenderness [21]. Conversely, bta-mir-182 may downregulate CAPN5, CASP2, and CASP9, which inhibits apoptosis. 

Calpain and calpastatin are associated with meat tenderness in beef cattle [21]. Calpain is a protease, which is initiated by calcium. Koohmaraie et al. [58] have proven that in postmortem muscles, cytoplasmic calcium concentration elevates slowly throughout the rigor mortis process, while the sarcoplasmic reticulum is unloaded [58]. This calcium translocation has an impact on the membrane permeability of this organelle, so as the binding of pro-apoptotic Bcl2 members [59]. The postmortem role of calpain is comprehensively studied, and the fact that this protein leads to meat tenderness is broadly accepted [60,61]. Calpastatin (CAST) is an inhibitor of calpain and the calpain: calpastatin ratio inversely correlates with tenderness in pork, lamb, and beef [62]. Calpastatin is a possible target for bta-mir-182, which indicates that elevated expression levels of this miRNA would impede the translation of the CAST gene, leading to decreased suppressive effects on calpain, and consequently a higher proteolytic activity postmortem and to increased meat tenderness [21]. A study conducted on Japanese black cattle determined another gene that was used as a target gene by bta-mir-885, the MTDH gene, that was found to be only expressed in the semitendinosus muscle (STD) in comparison to masseter muscle (MS). Possible functions of the MTDH gene are linked with the development of skeletal muscles, and it also has a regulative role in the transcription process [63].

### 3.8. Skeletal Muscle-Derived Satellite Cell miRNA

Skeletal muscle-derived satellite cells (MDSCs) regulate muscle growth, can improve muscle quality, and are primarily associated with healing of muscle [14]. Throughout the prenatal stage of livestock, muscle fibers are completely formed. During differentiation to MDSC, firstly, the progenitor cells proliferate and leave the cell cycle. Afterwards, they differentiate, align, and fuse to form myotubes [64]. The mentioned mechanism is controlled by myogenic regulatory factors and miRNAs.

**Figure 3:**
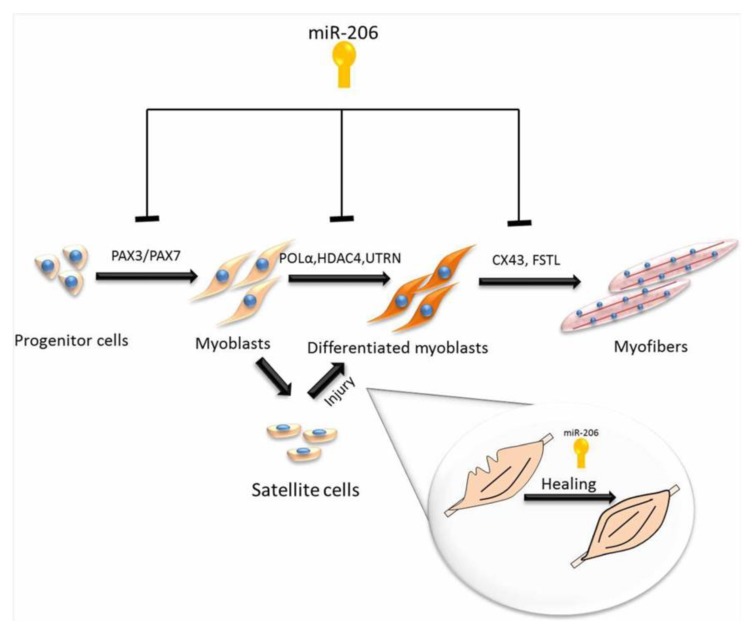
Roles of microRNA-206 in muscle regeneration and development.

By using high-throughput sequencing, Sun et al. [13] determined novel miRNAs from the longissimus thoracis of Chinese Qinchuan cattle [13]. Studies have identified that myogenic differentiation is controlled by muscle-specific miRNA, and include miRNAs such as miR-1 and miR-206, which stimulate myogenesis by upregulating the differentiation of satellite cells [65,66], while miR-133 participates in the proliferation process [67]. Using DNA sequencing to compare to known miRNAs, it has been determined that the muscle-specific miR-206 is the main miRNA in MDSC samples with more than 37.39% of total miRNAs in MDS C-D3 and 10.15% in proliferating satellite cells. The roles of miR-206 in muscle regeneration and development are summarized in Figure 3.

Unlike MDSC-D3, lower levels of miR-133 were determined in MDSC-D1, indicating that miR-206 and miR-1 play a more important part in the differentiation of MDSCs than miR-133 [68]. The expression level of miR-27b was 1.43- and 1.48-fold lower in MDSC-D1 and MDSC-D3, compared to MDSC-P. Myoblast proliferation is the result of the expression of miR-27b, which is related to decreased myostatin expression levels by targeting the 3’-UTR [36]. Recent evidence claims that during differentiation, miR-27b is highly expressed in satellite cells in mice [12]. However, in different muscle types and animal species, the molecular role of miR-27b can be changed.

Apart from muscle-specific miRNAs, several ubiquitous miRNAs could also have their roles in in muscle satellite cells. Bta-miR-99a-3p, bta-miR-2396, bta-miR-139, and bta-miR-10a were more abundant in MDSCs compared to MDSC-P. It is observed that miR-99a and miR-139 are associated with growth inhibition and apoptosis initiation, and miR-10a associated with the stimulation of the differentiation process [69]. By targeting Bcl-2, the upregulation of miR-181a in MDSC-D1 stimulated apoptosis [70], while targeting the MAPK-Snai2 pathway repressed tumor growth [71]. The miR-503 inhibited cell proliferation and cell cycle progression, but when targeting cyclin D1, this miRNA stimulated cycle quiescence [72]. 

Wong et al. [73] showed that 143 miRNAs presented a 2-fold lower abundance in MDSC-D1 compared to MDSC-P, e.g., miR-184 (log2 (MDSC-D3/MDSC-P) = –2.29) led to cell proliferation by targeting c-Myc [73]. Yet, the function of the mentioned miRNAs in the MDSCs differentiation is not completely identified. In a study conducted by Zhang et al. [14], a total of 617 miRNAs, including 53 novel candidates, were determined in comparison with those in MDSC-P: nine were up-expressed, 165 down-expressed, and 15 up-expressed, as well as 145 were down-expressed in MDSC-D1 and MDSC-D3. In MDSC-D3, compared to those in MDSC-D1, 17 up-expressed and 55 down-expressed miRNAs were determined [14]. All identified miRNAs are part of 237 gene families. Some sequences and base edits of the miRNAs were also detected. A detailed analysis of GO and KEGG pathways displayed that most target genes that were regulated by miRNAs had functions in cell processes and cancer pathways, as regulators of the actin cytoskeleton and the MAPK signaling pathway. In association with the 53 novel miRNAs, there were 7 up-expressed, 31 down-expressed, and 8 up-expressed, as well as 26 down-expressed in MDSC-D1 and MDSC-D3, compared to those in MDSC-P. Further, the expression levels of the 12 selected miRNA genes using RT-qPCR were compatible with the ones gained with deep sequencing.

## 4. Conclusions

This review has provided a summary of the current knowledge of the mechanism of miRNAs in relation to muscle tissue development and their potential effects on carcass and meat quality. The review provides molecular evidence to support the notion of several miRNAs directly influencing the differentiation of adipocytes by targeting different genes in cattle. With the increased application of technology in the field of meat quality and animal breeding programs, miRNAs are likely to be considered for the genetic advancement of beef quality.

Furthermore, the present review reflects on specific miRNAs expression patterns throughout muscle proliferation and differentiation, cell cycle progression, apoptosis, and metabolic pathways. Further experimentation and validation would be needed to prove the potential role and action mechanism of miRNAs in beef cattle for the purpose of carcass development and meat quality. Studies aimed at refining the knowledge of the miRNAs mechanism of action remains of pivotal importance for developing applications of miRNAs types, which will be valuable for marker-assisted selection in beef breeds.

## Figures and Tables

**Table 1:** The overview of microRNAs (miRNAs) associated with muscle functions.

Study	Tissue	miRNA
[18]	Intramuscular fat (IMF) deposition	miR-224
[24]	Adipose tissue	miR-23a~27a~24-2 cluster
[15]	Muscle	MEG3-DIO3 miRNA cluster (miR-758 and miR-543)
[19]	Intramuscular fat (IMF) deposition	Bta-miR-130
[11]	Various organs	miR-148a-3p
[21]	Longissimus thoracis (LT) muscle	350
[23]	Skeletal muscle and liver	bta-miR-486, bta-miR-7, bta-miR15a, bta-miR-21, bta-miR-29, bta-miR-30b, bta-miR-106b, bta-miR-199a-3p, bta-miR-204, and bta-miR-296
[12]	Skeletal muscle	miR-1, -128a, -133a, -133b, -139, -206, -222, -486, and -503
[14]	Skeletal muscle	617
[20]	Fetuses	bta-miR-23a
